# Cancer Patients' Perception, Acceptance, and Utilization of Artificial Intelligence‐Based Emotional Distress Assessment Tools: A Scoping Review

**DOI:** 10.1002/cam4.71538

**Published:** 2026-02-12

**Authors:** Carlos F. Urrutia, Joan C. Medina, Williams Contreras, Tania Estapé

**Affiliations:** ^1^ eHealthLab Universitat Oberta de Catalunya Barcelona Spain; ^2^ Department of Psychology and Education Sciences Universitat Oberta de Catalunya Barcelona Spain; ^3^ Institut Català d'Oncologia Barcelona Spain; ^4^ Child Tech Lab Universitat Oberta de Catalunya Barcelona Spain; ^5^ FEFOC Fundació Barcelona Spain

**Keywords:** acceptance, artificial intelligence, cancer, distress, mental health, oncology, perception, psycho‐oncology, screening, utilization

## Abstract

**Objective:**

Emotional distress in cancer patients and survivors impacts overall well‐being and quality of life. Several barriers to adequate screening have been identified and are currently being addressed by artificial intelligence (AI)‐based tools. However, there is a critical need to explore cancer patients' and survivors' perspectives on these new technologies. This scoping review aims to synthesize the available evidence on their perception, acceptance, and utilization of AI‐based voice, speech semantics, and facial expression (AIVSFE) tools for emotional distress screening.

**Methods:**

A systematic search was conducted in Scopus, Web of Science, PubMed Central, Cochrane Central Register of Controlled Trials (CENTRAL), PsycINFO, and Epistemonikos on July 1, 2025. Empirical studies published from January 1, 2019, to the search date that focused on adult cancer patients at any stage of treatment or survivorship and their perception, acceptance, or use of AIVSFE tools were retrieved. Participant sociodemographics, AI‐based distress screening modalities, technological frameworks, measurement tools, outcomes, and the studies' methodological quality were analyzed.

**Results:**

Three studies met the eligibility criteria. They included a combined sample of 316 cancer patients and survivors with heterogeneous clinical characteristics. Two studies utilized speech semantics technologies, while one utilized facial expression technology. The results show high acceptance, satisfaction, and usefulness rates (70%–98%), suggesting AIVSFE tools could address barriers associated with traditional distress screening.

**Conclusion:**

The findings indicate a favorable view of AIVSFE tools for detecting distress. Future studies should prioritize developing standardized evaluation frameworks, diversifying participant demographics, and addressing broader usability and ethical concerns to ensure equitable adoption of these technologies.

## 1 Introduction

Emotional distress poses a significant burden for cancer patients and survivors, impacting their overall well‐being [1, 2]. Left untreated, it can lead to poorer quality of life, treatment adherence, and medical outcomes [3]. Consequently, distress screening has been identified as a vital step in optimizing patient care outcomes and is recognized as an essential component of comprehensive cancer care [4, 5].

Definitions and measures of cancer‐related distress vary widely, resulting in heterogeneous screening tools [6]. Research has historically quantified emotional distress by identifying mental health symptoms such as anxiety or depression [7, 8]. However, these studies often overlook a significant proportion of people who experience cancer‐specific distress but do not meet the criteria for a formal mental health diagnosis, or do not have access to mental health professionals for such a diagnosis [9]. Studies have reported a range of modalities for measuring emotional distress, including validated scales, individual questions from validated questionnaires, and ad‐hoc distress instruments, as well as lifetime mental health diagnoses, posing challenges for proper distress management [10, 11].

Even if agreement could be reached on which measure to use, several barriers to implement successful emotional distress screening remain [12]. First, healthcare providers often lack the time or sufficient expertise to accurately assess and intervene with psychological distress [13]. Second, psychological distress surveys (either paper‐and‐pencil or digital) are often cumbersome to administer during a patient's visit, and many patients find completing the same survey multiple times repetitive and taxing [14, 15]. Finally, even in cancer centers that have successfully implemented distress screening protocols, assessments tend to be one‐time efforts and do not monitor the trajectory of psychological distress longitudinally. This cross‐sectional approach is problematic because cancer patients' distress trajectories fluctuate over time as they encounter different clinical phases—from the diagnosis and coping with treatment to survivorship and the fear of recurrence [12, 16]. These distinct phases trigger fluctuating psychological states that a single screening event can miss.

The failure to detect distress fluctuations is critical because effective treatments are available. Meta‐analyses have demonstrated that psychological interventions (e.g., Cognitive Behavioral Therapy, Mindfulness‐based therapies) significantly reduce distress and improve quality of life in cancer populations [17, 18]. However, the efficacy of these interventions is nullified if the patient's distress is never identified. Consequently, when screening fails or occurs too late, patients lose access to these evidence‐based support systems.

Recent advances in artificial intelligence (AI)‐based distress assessment tools focusing on language, speech, or facial expressions are being tested to address the above challenges [19, 20]. Spontaneously generated cues to psychological states and their recognition by AI can lead to improved screening for emotional distress [21]. More specifically, trimodal approaches (i.e., language, speech, and facial expression) have the potential to improve the accuracy of predictive models and reduce the likelihood of misclassifying signs of emotional distress [22].

As with any emerging technology, the effective adoption of AI‐based voice, speech semantics, and facial expression (AIVSFE) tools requires a fundamental understanding of users' willingness to accept and use them [23, 24]. In this context, acceptance implies the utilization of an intervention, as expressed in uptake rates, adherence, or satisfaction [25]. However, cancer patients' and survivors' prospective perception (i.e., future expectations), acceptance, and utilization of AIVSFE tools remain largely unexplored [20].

The aim of this scoping review is to synthesize, for the first time, the available evidence on perception, acceptance, and utilization of AIVSFE tools for detecting emotional distress in cancer patients and survivors. This synthesis would offer a valuable contribution to inform and guide current implementation efforts in this innovative field. Furthermore, the findings can shed light on trends in user perception during these early stages of AI adoption. It is hypothesized that the novel nature of the technology may result in limited awareness, which in turn can lead to mixed results, including both its potential clinical and operational advantages, as well as its associated risks.

## 2 Methods

**FIGURE 1:**
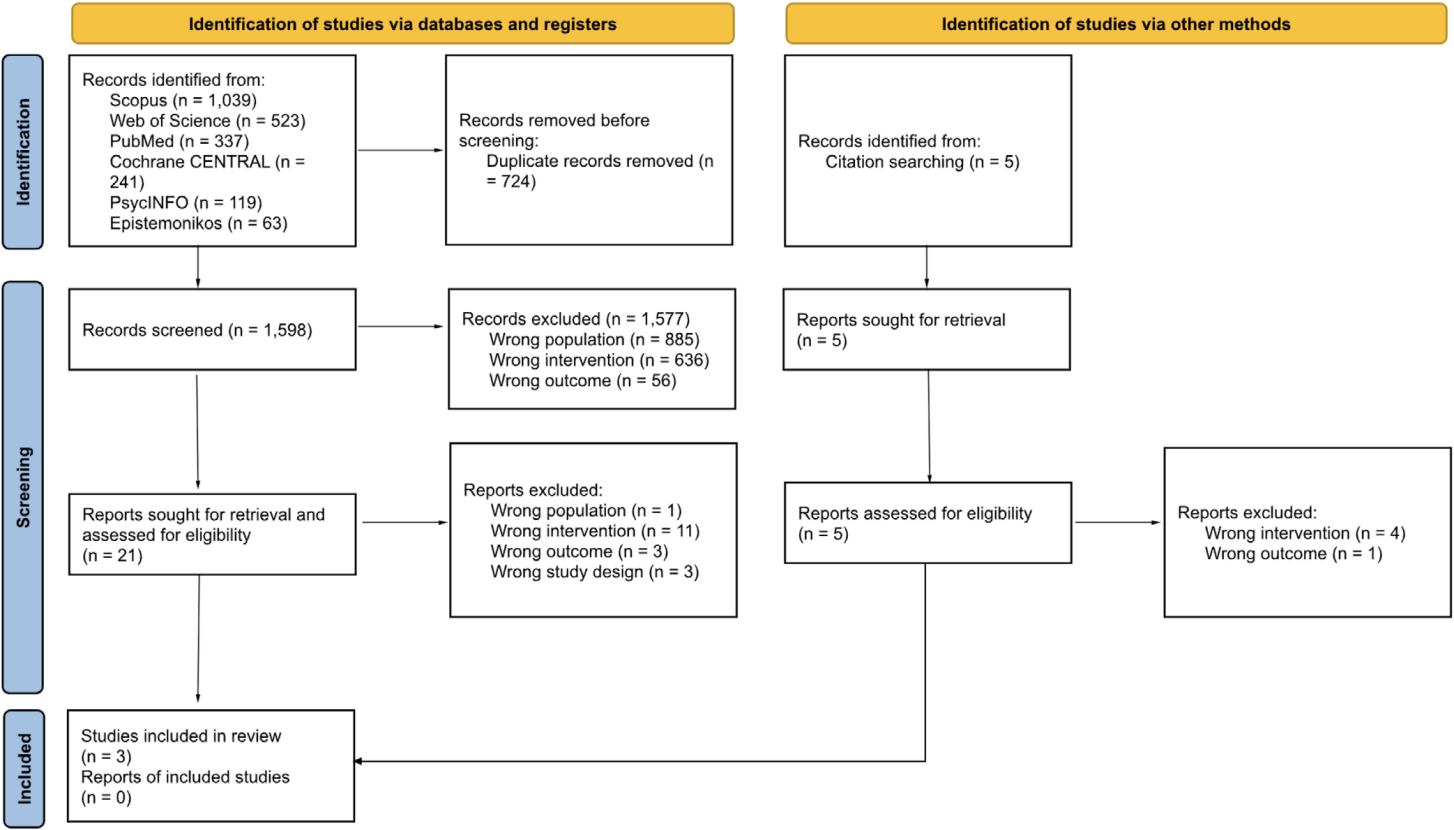
Flowchart of article selection according to PRISMA guidelines.

This scoping literature review was conducted according to the PRISMA Extension for Scoping Reviews (PRISMA‐ScR) guidelines [26] (see flowchart in Figure 1). The protocol was prospectively registered in PROSPERO (CRD42024509595).

### 2.1 Eligibility Criteria

Inclusion criteria were empirical studies that (a) reported quantitative or qualitative data, published between 2019 and 2025; (b) involved adult (≥ 18 years) cancer patients at any stage of treatment or survivorship; (c) described their perception, acceptance, or utilization of AIVSFE screening tools for emotional distress; and (d) were published in English or Spanish. Exclusion criteria were (a) studies involving child or adolescent cancer patients or survivors, and (b) palliative care studies at the end‐of‐life phase. The focus on the adult population stems from the understanding that this demographic group exhibits distinct characteristics and needs that differ from those of other younger age groups or advanced clinical stages. Moreover, the search for documents since 2019 is due to our interest in the most recent evidence in this novel topic. Studies were grouped according to the AIVSFE tools utilized.

### 2.2 Information Sources and Search Strategies

An initial search was conducted on May 2, 2024, and a subsequent search was conducted on July 1, 2025, with the objective of updating the results. In the initial search, documents from January 1, 2019, to the first search date were retrieved. In the subsequent search, documents were retrieved from May 1, 2024 to June 30, 2025. The Scopus, Web of Science, PubMed, Cochrane Central Register of Controlled Trials (CENTRAL), PsycINFO, and Epistemonikos databases were searched for publications.

The search process entailed using multiple controlled vocabulary terms and keywords for each concept of the topics under review, and then combining terms for each concept with Boolean operators. A representative but not inclusive list of terms for each concept are [1] Perception: satisfa* OR *advantag* OR barrier*; AND [2] Artificial Intelligence: “artificial intelligence” OR ai OR “machine learning”; AND [3] Cancer: onco* OR cancer OR tum?r*; AND Emotional Distress: “mental health” OR supportive OR psycho*. Searches were performed across subject headings, keywords, titles, and abstracts. The actual strategy, listing all the search terms used and how they were combined, is available in the Appendix.

### 2.3 Selection and Data Collection Processes

After establishing the inclusion and exclusion criteria, duplicates were removed using the Rayyan systematic review software [27]. The same software was used to review the retrieved reports through a stepwise procedure. The first stage of review focused on the title, abstract, and keywords and was conducted by two blind researchers. At this stage, interrater reliability was high (*κ* = 0.815, *p* < 0.001). In the second stage, full‐text review was completed, again by two blind researchers, with a senior author's support in case of disagreement. This time interrater reliability was moderate (*κ* = 0.481, *p* = 0.027). Where there was disagreement, consensus was reached through discussion. The Kappa index was computed using the “irr” package [28] for “R” software [29]. After review, backward and forward citation tracking was performed on the included reports, but no other publications were found to meet the eligibility criteria.

Data extraction and curation for included reports was conducted by one of the researchers using an Excel spreadsheet. Article outcomes were collected and analyzed according to this review's objectives, specifically cancer patients' and survivors' perception, acceptance, or utilization of AI‐based emotional distress tools. Additionally, participants' sociodemographic and clinical data were retrieved from the articles and analyzed.

Finally, article quality was assessed using the quality assessment tool for quantitative studies (QATQS) [30]. Two blind reviewers participated in this process, and discrepancies were resolved by consensus. Interrater reliability was substantial this time (*κ* = 0.622, *p* < 0.001). Studies were classified according to the AIVSFE features to detect emotional distress.

## 3 Results

### 3.1 Study Selection

A total of 1598 abstracts were identified from six databases and screened against our inclusion criteria. From this pool, 21 records were retrieved from the database and registry searches, and five records were retrieved via citation searching. Three articles were included in the final review [31, 32, 33]. The publication dates for the included articles ranged from 2022 to 2024.

The primary reasons for excluding reports in the first stage of review were related to populations (i.e., chronic diseases other than cancer; psychiatric and neurological diseases; children and adolescents; palliative cancer care), interventions (i.e., AI used to predict, diagnose, assess, or treat strictly oncological medical conditions; pharmacological interventions; tools other than AIVSFE), and outcomes (i.e., technical results from retrospective databases; healthcare and patient communication; patient and caregiver needs) beyond the scope of this review.

In the second stage, reports were excluded because interventions (i.e., decision tree chatbots, cloud‐based assistants, questionnaire‐based procedures), outcomes (i.e., clinical decision‐making, clinical team perceptions), and study designs (i.e., protocols, technical reporting) did not meet our eligibility criteria.

### 3.2 Study Characteristics

The three selected studies were from Western countries: two from Europe and one from North America. All articles used quantitative methods (i.e., ad‐hoc surveys and scales) to assess their results. The samples ranged from 48 to 166 patients, for a combined total of 316. The demographic composition of these samples was 67% female and 33% male. Education level was not reported in any of the three studies.

In terms of clinical characteristics, the samples' aggregated clinical data composition was 35% breast cancer, 32% hemato‐oncological cancer, 27% colorectal cancer, 1% gynecological cancer, 1% head and neck cancer, and 4% other types of cancer. Regarding the samples' clinical stages, 60% focused on the survivorship stage, 3% on the treatment stage, and 38% were not reported (see Table 1).

**TABLE 1:** Selected studies exploring cancer patients' and survivors' perceptions of multimodal technologies.

Study	Country	Participants	Illness data	AI distress modalities	Tools utilized	Outcomes	Quality assessment
Leung et al. [31]	Canada	48 Breast (50%) Colorectal (10%) Gynecological (6%) Head and neck (6%) Other cancers (25%) Unknown (2%)	Active treatment (17%) Posttreatment (46%) Other (37%)	Speech semantics	Artificial Intelligence–based Co‐Facilitator	Usefulness of AI‐provided resources (75%)	Weak
Safran et al. [32]	Belgium, Latvia, Slovenia, Spain	166 Breast (51%) Colorectal (49%)	Cancer survivors (100%)	Speech semantics	Multilingual Framework for Risk Assessment and Symptom Tracking	App features: Questionnaire user experience (75%) mHealth user experience (76%) Diary recording user experience (70%)	Weak
Wittenberg et al. [33]	Germany	102 Hemato‐oncologic cancer (100%)	Not reported	Facial expression recognition	Sophisticated High‐speed Object Recognition Engine	Meaningfulness of AI emotions analysis (88%) Not stressed by AI use (98%)	Weak

Quality assessment was performed according to QATQS guidelines. All studies received an overall rating of weak. Regarding component quality ratings, all had moderate selection bias, two had a moderate study design, and one had a weak study design [33]. All studies had moderate selection bias since the participants were sourced in a systematic manner yet not randomized. Regarding the study's design, the absence of randomized or controlled clinical trials resulted in the allocation of moderate and weak ratings.

All studies had weak confounders' control, two had moderate blinding, and one had weak blinding methods [32]. A confounder is a variable that is associated with an intervention or exposure and is causally related to an outcome of interest. It was not reported if control of the confounders in the study design or in the analysis had been implemented. Regarding blinding methods, the participants of two studies were not aware of the research question, resulting in a moderate rating for those studies. In contrast, one study did not disclose the methods employed for blinding.

Lastly, one study had strong data collection [32] and two had weak data collection methods. One study reported the temporal framework and outcomes collected throughout the study, whereas the other two did not address content reliability in their data collection methods. One had weak withdrawal and dropout management [32], while the other two were not assessable in this regard. Withdrawal and dropout management entails reporting the number of withdrawals and dropouts, as well as the reasons for them, if applicable.

### 3.3 Perception, Acceptance, and Utilization of AI‐Based Emotional Distress Tools

#### 3.3.1 Speech Semantics Methodologies and User Feedback

The multilingual framework for risk assessment and symptom tracking (MRAST) [32] and the artificial intelligence‐based co‐facilitator (AICF) [31] are innovative approaches to harnessing speech data to improve patient care. The MRAST prompts patients to record video diaries that capture their symptoms and experiences in real time. The framework utilizes an automatic speech recognition system to analyze the video recordings and extract symptoms and relevant information from the patients' spoken words, enabling a detailed understanding of their health status. Additionally, natural language processing (NLP) techniques are used to process the extracted data and identify symptoms and possible causes. An ad‐hoc Likert scale from 1 (poor) to 10 (excellent) was used to collect user feedback about the MRAST at three points in time (i.e., at the start of the app‐based questionnaire, after the introduction of the virtual agent, and at the end of the study). Overall usability received an average rating of 76%, their diary recording experience 70%, and their experience with structured questionnaires 75%.

The AICF was developed to identify keywords that indicate psychosocial concerns and to recommend appropriate online resources to members of an online support group (OSG) who express such a concern. The model was trained using a corpus of chat sessions from the CancerChatCanada (CCC) OSG and feedback from human annotations, providing language and expression resources used by cancer patients. The AICF utilizes an NLP model to create vector representations of terms and phrases related to psychosocial concerns expressed by participants. Once these concerns have been identified, the AICF uses a matrix to match these concerns with appropriate online resources, taking into consideration the patient's specific attributes. An ad‐hoc binary scale (“useful” and “not useful”) was used to collect participant feedback on this approach. Of the participants who accessed the resources recommended by the AICF, 75% rated them as “useful.” Therefore, although only two studies were considered, results suggest that AI‐based tools analyzing speech semantics are considered useful by most participants, leading to positive experiences.

#### 3.3.2 Facial Expression Methodology and User Feedback

The sophisticated high‐speed object recognition engine (SHORE) [33] software employs an object recognition engine to obtain patient data through real‐time emotion recognition. SHORE analyzes facial expressions based on underlying action units (AUs), which are then classified into basic facial emotions such as “happy,” “angry,” “sad,” “surprised,” and “neutral.” The software utilizes a camera‐based video stream to capture images of individuals in a separate room within the oncology outpatient clinic where participants have been invited to perform the experiment. An ad‐hoc questionnaire on meaningfulness and stressfulness was used to gather feedback from users: 98% of participants reported that they did not find the real‐time emotion analysis to be stressful, and 88% considered the real‐time analysis of their emotional state while waiting to be meaningful. No significant differences in acceptance were reported according to age or gender.

## 4 Discussion

The utilization of multimodal technologies for the detection of emotional distress in oncology populations is in the nascent stages of research. Although the initial phase of the review encompassed a substantial corpus of literature in the clinical field, a significant proportion centered on the application of AI in the diagnosis, prediction, and medical treatment of oncological diseases. In the engineering arena, conversely, others focused on presenting the technical outcomes of database implementations to assess the suitability of the interventions. In the full‐text review phase, we excluded articles that presented multimodal intervention projects in the oncology population but had not yet reported any outcomes or were still in the preliminary stages [34, 35, 36].

In total, two speech semantics technologies and one facial expression technology (all distress related) were selected for the analysis. Therefore, speech analysis, either speech‐to‐text or text direct, predominated in the selected studies. Conversely, no studies related to voice modality analysis (i.e., vocal biomarkers) were included in the review. It should be noted that some ongoing studies include voice analysis in their protocols [34, 36], so we can expect further exploration of its capabilities in the future. In any case, our findings so far are consistent with previous studies reporting that most existing tools use only one type of speech data: either voice or text, but not both [21, 37]. Conversely, studies on facial expressions were scarcely referenced in this review. Recent studies have reported the current challenges of transferring this modality from controlled laboratory environments to in‐the‐wild scenarios, which may be an optimal approach to effectively analyze emotional distress [38, 39]. This modality is still at an early stage of application, and more evidence is needed before any conclusions can be drawn.

Ethical risks associated with multimodal analysis need to be deeply addressed when assessing acceptance and use, especially in certain oncology settings like semi‐public spaces. Other perceived risks such as surveillance, informed consent, or intrusiveness have the potential to be heightened. Wittenberg et al.'s [33] data suggest that patients may not perceive immediate stress from monitoring in waiting rooms. However, the inherent vulnerability of this population requires that surveillance's intrusiveness be continuously weighed against clinical benefits to avoid inadvertently adding to the burden of care. To this end, the adoption of responsible AI policies is essential to ensure transparent data governance (i.e., explaining clearly how data are captured, anonymized, and stored) to safeguard patient rights and reduce perceived risks.

The characteristics of the study populations show some heterogeneity, with five cancer sites included (breast, colorectal, hemato‐oncological, gynecological, and head and neck). Furthermore, the reported phases include patients in active treatment and survival, the latter being by far the most common (60%). While it is acknowledged that the design of technologies applied to the oncology population requires adaptation to the characteristics of each cancer sites and sociodemographic conditions [40], larger and more diverse cohorts are still needed to fully establish the feasibility of detecting distress through speech or facial expression analysis. It should also be noted that the age ranges examined in these studies do not encompass the full spectrum of the cancer population, with the mean age tending to be around 60 years. Furthermore, the education level of the participants was not informed, which could hinder the general adoption of these applications, given the fact that cancer affects individuals across the lifespan. Recent studies have identified higher education level and younger age as determinants of becoming a digital native, alongside other factors such as breadth of use and experience, gender, and income [41, 42].

As previously mentioned, all studies received a weak overall quality rating. The research designs encompassed a pilot study, a feasibility and acceptance study, and a prospective study. It should be noted that no comparison groups or randomized techniques were applied. Instead, the current focus is on smaller cohorts and single‐arm studies. In the future, researchers may find it advantageous to incorporate qualitative methodologies or more robust quantitative designs (e.g., randomized controlled trials, clinical trials) in their research studies.

The three articles reviewed employed different methods of measuring and categorizing outcomes to ascertain user perceptions. Leung et al. [31] utilized a binary scale to assess usefulness, Safran et al. [32] used a Likert scale to assess their app's usability, while Wittenberg et al. [33] employed an ad‐hoc scale to assess meaningfulness and potential stress triggered by the applied technologies. No qualitative methods were reported to further investigate user perceptions aside from these scales. The frequent use of custom surveys to measure technology acceptance has been reported in the past [43], suggesting a lack of standardized measurement tools and consensus among researchers, despite efforts to consolidate unified models [44]. This poses a severe limitation for the external validity of the results. It seems that researchers feel compelled to design their surveys in such a way as to obtain a measurement instrument adapted to the specific issues of their target population or with additional context‐specific constructs [45].

As for the terminology used to describe users' perspectives on AI tools, the studies we reviewed included a range of terms such as “usefulness,” “user experience,” and “meaningfulness.” Like custom surveying, this diversity in terminology illustrates the complexity of assessing technology acceptance [43, 46]. Although researchers aim to use established theoretical frameworks for assessments, they often rely on custom terminology and surveys, as seen in the selected studies. These ad‐hoc tools may enable researchers to tailor their investigations to specific contexts, but their use poses challenges for the comparability of findings across studies.

The perception, acceptance, and usability outcomes of these studies were positive, ranging from 70% to 98%. Specifically, one speech semantics study reported a perceived usefulness of 75% [31], while the other reported its users' experience with the app's features, giving 75% for the questionnaire experience, 76% for mHealth, and 70% for diary recording [32]. The findings from both studies suggest the efficacy and user‐friendliness of speech‐based tools, with the caveat that improvements are recommended, particularly in the diary recording feature. The study employing facial expression modality reported that 88% of patients found it meaningful to have their emotions analyzed in the waiting room, and 98% did not experience any stress related to recording and analyzing their emotions [33]. These findings suggest that facial expression analysis in outpatient settings is preliminarily perceived as a feasible and useful method of emotional assessment that could save clinicians' time. Altogether, the participants in these studies responded positively, suggesting that AIVSFE technologies are potentially well perceived by patients in the context of cancer care. These results are similar to those reported quantitatively and qualitatively in systematic reviews focused on supportive care interventions in oncology patients [47, 48].

### 4.1 Clinical Implications

The transformation of emotional distress assessment models through AI has clinical and technical implications. From a clinical perspective, AIVSFE tools offer advantages in psychological distress assessment by addressing the limitations of traditional screening methods. These technologies have the potential to save time for both patients and healthcare professionals by automating distress detection and reducing the burden of constant self‐reporting. Unlike conventional survey‐based approaches, AI models can provide a more accurate and continuous evaluation by analyzing vocal, speech, and facial expression patterns in real time. Notably, embedded within the broader landscape of eHealth innovations, their ability to facilitate remote assessments reduces the need for in‐person visits, making psychological distress monitoring more accessible, particularly for patients facing mobility challenges or geographical barriers.

The implementation of AIVSFE tools has the potential to address the detection gap previously outlined. Unlike one‐time survey assessments, these tools enable continuous, passive monitoring. This capability is suited to capture the fluctuating distress trajectories associated with different clinical phases (e.g., diagnosis, treatment, fear of recurrence). By identifying these shifts in real‐time, AIVSFE tools can serve as a bridge to evidence‐based interventions, ensuring that patients are promptly connected to effective therapies that might otherwise remain underutilized due to insufficient or delayed detection.

However, the integration of AIVSFE tools into health care also presents challenges and ethical dilemmas. Patients who accept and use these tools may worry about the confidentiality of sensitive health information that is collected and stored as they interact with these systems. Ensuring the security and privacy of patient records is critical to complying with legal standards, requiring robust safeguards to prevent unauthorized access or disclosure [49]. These steps are essential to maintain trust and protect patients' rights in AIVSFE applications.

The present state of AIVSFE tools has not yet surmounted the inherent challenges that preclude their comparison with current emotional distress tools for oncology populations. Despite the potential benefits of these tools, including their increased ubiquity and reduced effort, further evidence from more robust study designs and replicable outcome measures is crucial to encourage their adoption by healthcare providers, users, and policymakers. The incorporation of responsible AI policies is also imperative for future clinical implementation and comparison with current distress tools.

### 4.2 Limitations and Needs for Future Research

There are several limitations to the review's findings. First, although every effort was made to cover the most recent evidence, this review is subject to continuous updating as new AI‐related models continue to develop. Second, there is a lack of consensus on the terminology and measurement methods among the reports retrieved by our search. Third, the samples included in the studies are small and heterogeneous, and at the same time, they focus on the survivorship phase or are in Western countries. Therefore, results cannot be generalized to all patients. Fourth, given the different characteristics and needs of pediatric, adolescent, and palliative care populations, the results of this study are not applicable to these cohorts. Consequently, their perceptions must be examined independently of adult perceptions to determine its applicability. Finally, the inclusion of studies with more robust methodological designs could lead to more replicable results in the future.

Literature on the acceptance and usability of multimodal models for detecting emotional distress in the oncology population is still emerging, which makes it difficult to draw definitive conclusions. However, this review highlights key findings and identifies critical limitations that can guide future research. By building on these insights, subsequent studies can address the gaps identified herein and move closer to resolving challenges such as sample heterogeneity, methodological variability, and the need for longitudinal data to assess the long‐term effectiveness of AIVSFE tools for screening emotional distress. Additionally, the technical complexities of developing AI models must be considered, particularly the risks of assessment biases and the lack of representativeness in training data sets, which may compromise the generalizability of these tools. Future research should prioritize the inclusion of diverse patient populations and establish rigorous validation frameworks to mitigate these risks. Furthermore, the substantial time and human resources required for training, retraining, and supervising must be considered.

## 5 Conclusion

This scoping review is the first to address patients' experience with multimodal technologies leveraging AI to detect emotional distress in oncology populations. Despite the limited number of studies that met the inclusion criteria, highlighting their early‐stage development, the findings underscore promising advances in the use of AIVSFE tools to assess and address patient needs.

Significant gaps in current research have been identified, including lack of consensus on outcome measures, reliance on ad‐hoc scales, and limited diversity in study samples. Nevertheless, these tools appear to be well accepted by patients and survivors, provided the identified limitations are resolved. This would open a range of possibilities given their advantages, such as time‐saving and accurate detection. Priorities for future studies should include developing standardized evaluation frameworks, diversifying participant demographics, and addressing broader usability and ethical concerns to ensure the equitable adoption of these technologies.

While the integration of AIVSFE tools with cancer patients and survivors shows initial potential for enhancing emotional distress detection and patient care, further research and collaborative efforts are essential to refine these tools and address the identified limitations. As AI‐driven solutions continue to evolve, their success will hinge on balancing technical innovation with patient‐centered design and inclusivity.

## Author Contributions

**Carlos F. Urrutia:** conceptualization (equal), data curation (equal), formal analysis (equal), investigation (equal), methodology (equal), writing – original draft (equal). **Joan C. Medina:** methodology (equal), resources (equal), supervision (equal), validation (equal), writing – review and editing (equal). **Williams Contreras:** formal analysis (equal), investigation (equal). **Tania Estapé:** methodology (equal), resources (equal), supervision (equal), validation (equal), writing – review and editing (equal).

## Funding

This work was supported by Universitat Oberta de Catalunya.

## Ethics Statement

This paper is part of a research project reviewed and approved by the Universitat Oberta de Catalunya's Research Ethics Committee (CE24‐TE05).

## Conflicts of Interest

The authors declare no conflicts of interest.

## Data Availability

The data analyzed in this manuscript are available upon reasonable request from the corresponding author.

## Appendix 1 Search Query

(TITLE‐ABS‐KEY (facilitator OR facilitation OR satisfa* OR *advantag* OR barrier* OR perce* OR accept* OR usage OR user* OR usab* OR utili?ation OR adopt* OR “planned behavio?r” OR “reasoned action” OR “motivational model” OR “innovation diffusion” OR “social cognitive” OR “performance expecta*” OR “effort expecta*” OR “social influence” OR appropriateness) AND TITLE‐ABS‐KEY (“artificial intelligence” OR ai OR “machine learning” OR “natural language processing*” OR “neural network*”) AND TITLE‐ABS‐KEY (onco* OR cancer OR tum?r* OR malignan* OR neoplas* OR *carcino* OR leukemia OR leukaemia OR lymphoma OR myeloma OR metasta* OR melanoma OR sarcoma) AND TITLE‐ABS‐KEY (“mental health” OR supportive OR psycho* OR stress* OR anxi* OR depress* OR distress*)) AND PUBYEAR > 2018 AND (LIMIT‐TO (LANGUAGE, “English”) OR LIMIT‐TO (LANGUAGE, “Spanish”)).
